# Longitudinal Association of Maternity Care Practices with Exclusive Breastfeeding in U.S. Hospitals, 2018–2022

**DOI:** 10.3390/children12111454

**Published:** 2025-10-26

**Authors:** Lucas Gosdin, Kristin J. Marks, O. Yaw Addo, Lauren O’Connor, Sofia Awan, Daurice A. Grossniklaus, Heather C. Hamner

**Affiliations:** 1 Division of Nutrition, Physical Activity, and Obesity, National Center for Chronic Disease Prevention and Health Promotion, Centers for Disease Control and Prevention, Atlanta, GA 30341, USA; 2 United States Public Health Service, Rockville, MD 20852, USA; 3 Oak Ridge Institute for Science and Education, Oak Ridge, TN 37831, USA

**Keywords:** breastfeeding, maternity care, exclusive breastfeeding, hospital support

## Abstract

**Highlights:**

**What are the main findings?**

• Certain maternity care practices and policies can support families to breastfeed.
• Across the United States, hospitals that improved and sustained maternity care practices were more likely to have higher in-hospital exclusive breastfeeding rates.

**What is the implication of the main finding?**

• Improving and sustaining maternity care practices and policies supportive of breastfeeding might increase in-hospital exclusive breastfeeding over time.

**Abstract:**

**Background/Objectives**: Breastfeeding has health benefits for infants and mothers, and hospitals play an important role in supporting breastfeeding. This analysis examines the longitudinal association of hospital maternity care practices and policies with in-hospital exclusive breastfeeding rates from 2018 to 2022. **Methods**: U.S. hospitals completing ≥2 surveys during 2018, 2020, and 2022 cycles of CDC’s Maternity Practices in Infant Nutrition and Care (mPINC) survey comprised a nested longitudinal cohort (n = 2109). Hospitals were given a modified mPINC score (0 to 100 points) based on self-reported adherence to maternity care practices and policies supportive of breastfeeding, including skin-to-skin contact, monitoring following birth, rooming-in, feeding counseling and education, and institutional policies. Hospitals reported their exclusive breastfeeding rates for healthy infants for the duration of hospitalization. A path analysis quantified the total effects of modified mPINC scores on in-hospital exclusive breastfeeding rates in subsequent survey cycles, controlling for annual births. **Results**: Among hospitals with the highest modified mPINC scores of 100 points, the mean in-hospital exclusive breastfeeding rates were 62.0% in 2018 (n = 129), 62.2% in 2020 (n = 132), and 61.7% in 2022 (n = 138). Hospitals with the lowest scores of <60 points had exclusive breastfeeding rates of 40.6% (n = 247), 41.9% (n = 173), and 37.8% (n = 127), respectively. Hospitals that increased their modified mPINC score by 10 points from 2018 to 2022, regardless of their score in 2018, had a 2.0 p.p. increase in their exclusive breastfeeding rates. In an adjusted path analysis, each 10-point higher modified mPINC score in 2018 was associated with a 4.4 (95% CI, 4.0–4.9) percentage point higher exclusive breastfeeding rate in 2022—through increasing the likelihood of higher in-hospital exclusive breastfeeding rates in 2018 and 2020 and higher modified mPINC scores in 2020 and 2022. **Conclusions**: Improving and sustaining maternity care practices and policies supportive of breastfeeding are associated with higher in-hospital exclusive breastfeeding over time.

## 1. Introduction

Breast milk is the optimal form of nutrition for most infants and has health benefits for children and women who breastfeed them [1,2,3,4]. While most families in the United States initiate breastfeeding (84.1% for children born in 2021) [5], fewer achieve recommendations to exclusively feed breast milk through about 6 months of age [6,7,8] (27.2% for children born in 2021) [5]. Families are more likely to provide breast milk to their children when they are supported to do so [9]. Because around 98% of deliveries in the United States are in hospitals [10], they are the first environment where most infants are fed and are important partners in supporting families to start breastfeeding.

Certain maternity care practices are associated with better breastfeeding outcomes including in-hospital exclusive breastfeeding [9]. The World Health Organization (WHO) and United Nations Children’s Fund (UNICEF) established the Baby-Friendly Hospital Initiative to implement a collection of hospital practices and policies supportive of breastfeeding called the Ten Steps to Successful Breastfeeding (Ten Steps) [11].

Various cross-sectional studies have found that hospitals with maternity care practices and policies supportive of breastfeeding are more likely to have higher in-hospital exclusive breastfeeding rates [12,13,14]. Prospective studies in the United States have also found that individuals who receive maternity care consistent with the Ten Steps have better breastfeeding outcomes [13]. Other studies have examined the effects of quality improvement initiatives in hospitals and found that improving maternity care practices and policies to align with the Ten Steps was associated with improved in-hospital exclusive breastfeeding rates [15,16]. However, there is limited evidence, representing the diversity of hospitals in the United States, that documents the effects of maternity care practices supportive of breastfeeding on hospital-level breastfeeding outcomes over time at the national level.

This analysis examines the longitudinal association of maternity care practices supportive of breastfeeding on in-hospital exclusive breastfeeding within diverse hospitals across United States and territories during 2018 to 2022.

## 2. Materials and Methods

The Centers for Disease Control and Prevention (CDC) conducts a biennial survey of Maternity Practices in Infant Nutrition and Care (mPINC) for which all hospitals that provided maternity care in United States and territories in the preceding year are eligible [17].

This analysis was limited to the survey cycles that occurred after it was revised in 2018 to capture more recent developments in infant feeding-related U.S. maternity care. Hospitals completing at least two surveys during the 2018, 2020, and 2022 survey cycles were included to form a nested longitudinal cohort (n = 2109).

In 2018, 2045 of 2913 eligible hospitals participated (70%), of which 1811 completed at least one subsequent survey cycle. In 2020, 2103 of 2810 eligible hospitals participated (75%), of which 1949 completed at least one additional survey cycle. In 2022, 1994 of 2779 eligible hospitals participated (72%), of which 1822 completed at least one previous survey cycle. There were 1521 hospitals that participated in both 2018 and 2022.

### 2.1. Measures

The mPINC questionnaire includes 21 measures of maternity care practices and policies that are consistent with national and international recommendations and supported by scientific evidence [18]. These measures are scored relative to best practices in maternity care and are sorted into domains and averaged. Then, the domain scores are combined into an overall score for each participating hospital [19]. Because this analysis focuses on in-hospital exclusive breastfeeding, practices that related directly to the outcome (proportion of breastfed newborns fed infant formula and the proportion of formula-fed infants whose parents were taught formula preparation and formula feeding techniques) or that occurred after the outcome (discharge support) were excluded from the exposure, resulting in a modified mPINC score. The remaining measures within the feeding practices and feeding education domains were combined after removing the excluded measures because they were conceptually similar. Combining these two domains also ensured that each domain has at least three component measures. The modified mPINC score consisted of 15 measures covering four domains: immediate postpartum care; rooming-in; feeding practices, education, and support; and institutional management (Table 1). The measures were scored within each domain by taking the mean of its component survey items, and the modified mPINC score is an average of the four domains. The modified score ranges from 0 points (implementing none of the 15 best practices and policies) to 100 points (fully implementing all 15 best practices and policies).

In-hospital exclusive breastfeeding rate was reported by each hospital as the percent of healthy newborns who received only breast milk—and no water or formula at any time during hospitalization as well as no glucose water or sucrose solution except during painful procedures. Hospitals reported either an actual percentage (51% of hospitals) or an estimated percentage (49% of hospitals) of in-hospital exclusive breastfeeding. Hospitals did not report how they arrived at estimates. Within each survey year, mean in-hospital exclusive breastfeeding was approximately 3 percentage points higher for those who reported an actual percentage than those who reported an estimated percentage.

### 2.2. Analysis

First, mean in-hospital exclusive breastfeeding rates are presented by modified mPINC domain scores and overall scores within each survey cycle. Since the mPINC survey is a census and, therefore, has no sampling error, descriptive statistics are presented without inferential statistics.

Second, to isolate the effects of changes in maternity care practices, an ordinary least squares model of differences was used to estimate the average change in in-hospital exclusive breastfeeding rate between 2018 and 2022 for each 10-point change in modified mPINC score over the 4-year period. A 10-point change was used because it is a familiar difference for a 100-point scale and represents implementation of one or two improved practices. This method quantifies the magnitude and direction of changes and treats no change in maternity care practices and exclusive breastfeeding rate as zero for the exposure and outcome, respectively.

Third, to assess the longitudinal association of both changing and sustaining higher mPINC scores with in-hospital exclusive breastfeeding rate, a multi-mediation structural equation model (path analysis), with full information maximum likelihood for missing data, was fit. This model estimated the direct, indirect, and total effects of modified mPINC score on in-hospital exclusive breastfeeding rates across time. Model fit was assessed by both omnibus—chi-square, root mean square error of approximation (RMSEA), and standardized root mean square residual (SRMR)—and incremental fit indices—comparative fit index (CFI) and Tucker-Lewis index (TLI) [20]. The final model was equivalent to the a priori model. Structural equation models can account for both changing and sustaining maternity care practices through multiple, simultaneous regression equations.

Finally, as a sensitivity analysis, a full structural equation model was fit to examine how the association might change if the modified mPINC score was substituted with a data-driven approach to combining the measured practices and policies. This model was equivalent to the path analysis except that the modified mPINC score was replaced with a latent factor (“maternity practices”) representing the 15 observed measures that make up the modified mPINC score. The measures were dichotomized based on the ideal response for each. Factor loadings for each observed measure were constrained across time so that latent factors were equivalent for each survey cycle. This model was refit to include covariance parameters among the endogenous variables of latent factors as suggested by modification indices and consistent with operating hypotheses [21].

All analyses were conducted in R version 4.2.1 (The R Foundation for Statistical Computing, Vienna, Austria) using the “tidyverse” and “lavaan” packages [21,22]. All models were adjusted for hospital size. Other variables were assessed for confounding, including hospital type, geographic region, and level of neonatal care, but did not change estimates or interpretation and were left out in favor of a more parsimonious model. An a priori alpha was set at 0.05 for statistical significance.

## 3. Results

Hospitals included in this nested cohort were diverse and geographically well distributed. Most hospitals were non-profit (77.1%), had fewer than 1000 annual births (56.7%), and did not have a neonatal advanced care unit (70.7%) (Table 2).

Overall, among hospitals with modified mPINC scores of 100 points (highest score), the mean in-hospital exclusive breastfeeding rates were 62.0% in 2018, 62.2% in 2020, and 61.7% in 2022; whereas hospitals with scores of <60 points (lowest scores) had in-hospital exclusive breastfeeding rates of 40.6%, 41.9%, and 37.8%, respectively (Table 3). In each survey year and within each domain of maternity care practices and policies, there was an increase in in-hospital exclusive breastfeeding rate with increasing modified mPINC score. For example, in 2022, hospitals with scores of <60, 60–79, 80–99, and 100 points in the Rooming-In domain had in-hospital exclusive breastfeeding rates of 49.1%, 52.9%, 53.6%, and 56.1%, respectively. Hospitals with scores of <60, 60–79, 80–99, and 100 points in the Feeding Practices, Education, and Support domain had in-hospital exclusive breastfeeding rates of 37.5%, 47.0%, 51.4%, and 56.7%, respectively.

**Figure 1:**
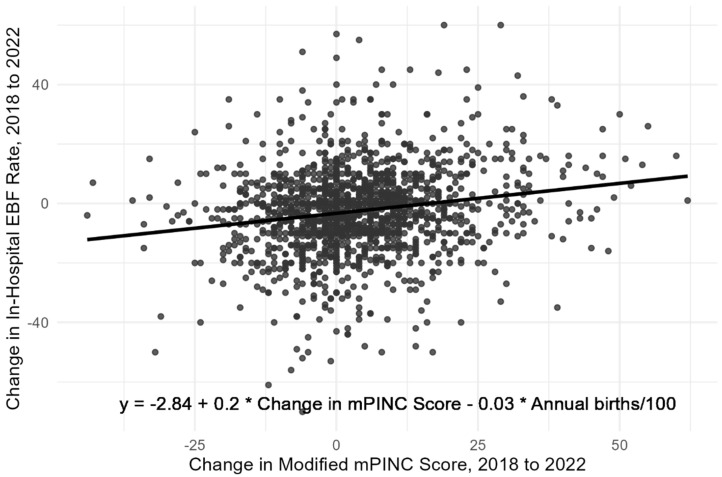
Change in rate of in-hospital exclusive breastfeeding (EBF) relative to the change in modified mPINC score—Maternity Practices in Infant Nutrition and Care (mPINC) Survey, United States, 2018–2022 (n = 1521) ^a^. Note: A 10-point increase in modified mPINC score between 2018 and 2022 was associated with a 2.00 (95% CI, 1.46–2.54) percentage point increase in in-hospital exclusive breastfeeding rate, controlling for the number of annual births in the hospital. ^a^ Limited to hospitals that participated in both 2018 and 2022 mPINC survey cycles.

In an ordinary least squares model of differences (n = 1521), a 10-point increase in mPINC score between 2018 and 2022 was associated with a 2.00 (95% CI, 1.46–2.54) percentage point (p.p.) increase in the rate of in-hospital exclusive breastfeeding during the same period, controlling for the number of births in the hospital (Figure 1).

**Figure 2:**
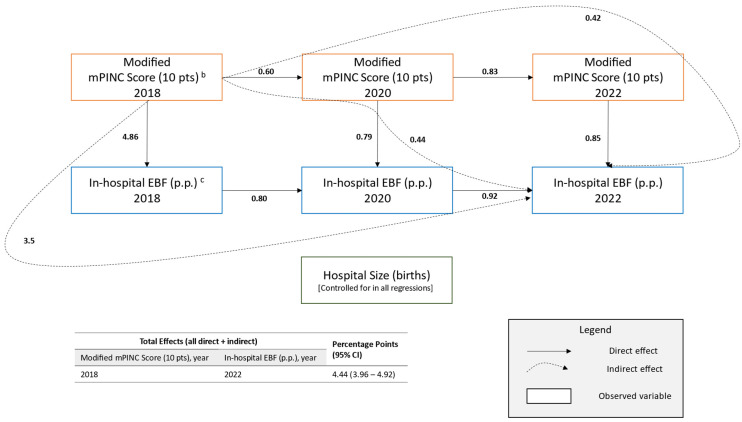
Path diagram of the multi-mediation model of maternity care practices and policies and in-hospital exclusive breastfeeding (EBF) rates—Maternity Practices in Infant Nutrition and Care (mPINC) Survey, United States, 2018–2022 (n = 2109) ^a^. Note: pts, points; p.p., percentage points; CI, confidence interval. Unstandardized estimates. Estimates are in units of the dependent variable. All paths *p* < 0.001. Model Fit Statistics: Chi-square (6 df) = 57.3 (*p* < 0.05); root mean square error of approximation = 0.064; standardized root mean square residual = 0.021; comparative fit index = 0.992; Tucker-Lewis index = 0.973. ^a^ Nested cohort of hospitals surveyed at least twice during the 2018, 2020, and 2022 cycles of the Maternity Practices in Infant Nutrition and Care (mPINC) survey. This model includes only those with data on the exogenous variable (2018 modified mPINC score). ^b^ Modified mPINC score calculated as the average of each of the domain scores. Not directly aligned with published mPINC scores. Domains include (a) immediate skin-to-skin contact, transition from delivery to rooming-in, and monitoring following birth; (b) rooming-in, mother-infant separation, and rooming-in safety; (c) glucose monitoring of healthy newborns not at risk of hypoglycemia, formula counseling for breastfeeding mothers, education on feeding cues and pacifiers, and education on how to identify and solve breastfeeding problems; (d) requiring nurse skill competencies, nurse competency assessment, documentation of exclusive breastfeeding, acquisition of infant formula, and written policies supportive of breastfeeding. ^c^ In-hospital exclusive breastfeeding is the percent of newborns who received only breast milk and no water or formula at any time during hospitalization as well as no glucose water or sucrose solution except during painful procedures.

In an adjusted path analysis (n = 2109), each 10-point higher modified mPINC score for a hospital in 2018 was associated with a 4.44 (3.96–4.92) p.p. (Figure 2) higher in-hospital exclusive breastfeeding rate in 2022 (standardized coefficient = 0.34 [0.30–0.37]; Table A1). This association was driven by increasing the likelihood of higher subsequent modified mPINC scores (indirect effect: 0.42 [0.20–0.65] p.p.), higher in-hospital exclusive breastfeeding rates in previous years (indirect effect: 3.58 [3.14–4.01] p.p.), and higher scores with higher exclusive breastfeeding rates in the intervening years (indirect effect: 0.44 [0.21–0.66] p.p.) (Figure 2).

**Figure 3:**
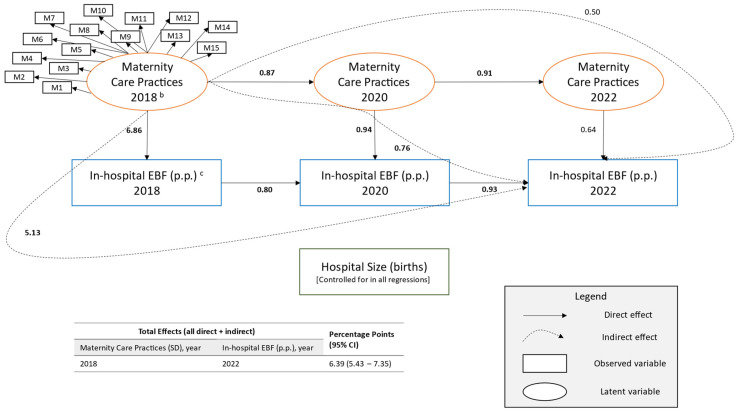
Path diagram of the structural equation model of maternity care practices and policies and in-hospital exclusive breastfeeding (EBF) rates—Maternity Practices in Infant Nutrition and Care (mPINC) Survey, United States, 2018–2022 (n = 2109) ^a^. Note: p.p., percentage points; SD, standard deviation; CI, confidence interval. Only latent variables are standardized. Estimates are in units of the dependent variable. All paths shown in bold *p* < 0.001. Model Fit Statistics: Chi-square (1011 df) = 3215.7 (*p* < 0.001); root mean square error of approximation = 0.032; standardized root mean square residual = 0.050; comparative fit index = 0.923; Tucker-Lewis index = 0.911. ^a^ Nested cohort of hospitals surveyed at least twice during the 2018, 2020, and 2022 cycles of the Maternity Practices in Infant Nutrition and Care (mPINC) survey. ^b^ M1 to M15 represent the measures of maternity care practices which include (a) immediate skin-to-skin contact, transition from delivery to rooming-in, and monitoring following birth; (b) rooming-in, mother-infant separation, and rooming-in safety; (c) glucose monitoring of healthy newborns not at risk of hypoglycemia, formula counseling for breastfeeding mothers, education on feeding cues and pacifiers, and education on how to identify and solve breastfeeding problems; (d) requiring nurse skill competencies, nurse competency assessment, documentation of exclusive breastfeeding, acquisition of infant formula, and written policies supportive of breastfeeding. ^c^ In-hospital exclusive breastfeeding is the percent of newborns who received only breast milk and no water or formula at any time during hospitalization as well as no glucose water or sucrose solution except during painful procedures.

Results were similar when the modified mPINC score was replaced with a latent variable of its component measures. In a full structural equation model (n = 2109), with each one standard deviation increase of the latent variable “maternity care practices” for a hospital in 2018 there was a 6.39 (5.43–7.35) p.p. higher in-hospital exclusive breastfeeding rate in 2022 (standardized coefficient = 0.30 [0.26–0.34]; Table A2). This association was also driven by indirect effects through increasing the likelihood of higher subsequent modified mPINC scores (indirect effect: 0.50 [−0.07–1.08] p.p.), higher in-hospital exclusive breastfeeding rates in previous years (indirect effect: 5.13 [4.33–5.92] p.p.), and higher scores with higher exclusive breastfeeding rates in the intervening years (indirect effect: 0.76 [0.22–1.29] p.p.) (Figure 3).

## 4. Discussion

Over a 4-year period, U.S. hospitals with better maternity care practices and policies supportive of breastfeeding had higher rates of in-hospital exclusive breastfeeding. For example, hospitals with 10-point higher modified mPINC scores in 2018 had 4.4 p.p. higher in-hospital exclusive breastfeeding rates in 2022 by increasing the likelihood of having higher subsequent modified mPINC scores and exclusive breastfeeding rates. Hospitals that increased their modified mPINC score by 10 points during the same period, regardless of their score in 2018, had a 2.0 p.p. increase in their exclusive breastfeeding rate. This shows that both sustaining and improving maternity practices supportive of breastfeeding can potentially result in improvements of in-hospital exclusive breastfeeding. For example, a 10-point increase in modified mPINC score, as examined in this analysis, is equivalent to implementing two policies in the institutional management domain or approximately one to two practices from the remaining domains. These policy or practice differences are associated with meaningful improvements for in-hospital exclusive breastfeeding.

Previous studies have found similar results when examining maternity care practices cross-sectionally with higher mPINC scores being correlated with higher rates of in-hospital exclusive breastfeeding [23]. Other evaluations have found that increasing hospital supports for breastfeeding were associated with increased breastfeeding rates. In one evaluation of a state quality improvement collaborative, hospitals that began implementing an average of 2.2 more of the Ten Steps over 2 years had approximately 17 p.p. higher in-hospital exclusive breastfeeding rates [24]. In an ecological analysis, a state’s average hospital mPINC score increased by 15 points over 4 years and coincided with an increase of 17 p.p. in the state in-hospital exclusive breastfeeding rate [15]. The aforementioned evaluations estimated larger effects than this analysis; however, they may not be directly comparable because they were done in the context of intensive interventions in smaller geographic areas. Data that are more directly comparable to the present analysis are limited.

This study is strengthened by national, hospital-level data that spans multiple years with robust and consistent response rates. The evidence-based practices and policies were assessed holistically, which more closely mirrors how they are implemented rather than examining individual practices. The study examined the effects associated with both changing practices and maintaining better practices, which also better reflects their implementation than examining changes alone.

### 4.1. Limitations

This study is also subject to several limitations. First, this is an observational analysis and, therefore, limits the ability to infer a causal relationship between the exposure and outcome. This design is also unable to account for changes in unobserved factors that might have affected exclusive breastfeeding rates during this period, such as changes in the patient population and other potential unmeasured confounding variables. Second, the mPINC survey is completed by the person(s) most knowledgeable of infant feeding in the hospital but might not reflect hospital practices and policies with complete accuracy. In-hospital exclusive breastfeeding is also reported by the hospital respondent as either the actual percentage or an estimate. It is unclear how hospitals arrive at their estimates and are likely subject to error. However, those reporting actual percentages had slightly higher proportions of in-hospital exclusive breastfeeding, which is expected given that tracking exclusive breastfeeding is part of the Ten Steps. Third, while the survey is a census, the response rates varied, and there may have been differential participation by hospital maternity practices and in-hospital exclusive breastfeeding rates. However, the nested cohort design limits the effects of any such biases since the longitudinal comparisons are made among the same group of hospitals over time. Fourth, the modified mPINC score, which treats each measure within a domain as equal, might not optimally capture maternity care practices and policies as they relate to in-hospital exclusive breastfeeding. However, the mPINC score is associated with breastfeeding outcomes and is an important tool for hospitals and public health surveillance [14,17]. Fifth, we removed measures of practices related to formula feeding and discharge, which further focused the modified mPINC score on practices and policies that could affect in-hospital exclusive breastfeeding. Thus, the modified score cannot be directly compared to the mPINC score.

Because of the potential limitations of the modified mPINC score, maternity care practices and policies were also modelled as a latent variable of its component measures using a full structural equation model. This did not change the pattern of the relationships observed, adding credibility to the modified mPINC score when examining in-hospital exclusive breastfeeding as an outcome. In fact, the standardized coefficients for the examined total effects are very similar.

Model fit indices indicated good fit for the path model [20]. The full structural equation model has only fair fit, but its composition is guided by causal hypotheses and was therefore not refit with additional parameters that veered from the hypothesized relationships. We interpret the full structural equation model as confirming the pattern seen in the path model.

### 4.2. Implications

This analysis observes improvements of in-hospital exclusive breastfeeding rates among hospitals with maternity care practices and policies supportive of breastfeeding—during a period of little to no improvement in mPINC scores at the national level [25]. This highlights opportunities to improve maternity care and infant nutrition.

## 5. Conclusions

These results can be used to build support for improving and maintaining maternity care practices supportive of breastfeeding, which can have lasting effects on breastfeeding outcomes in hospitals. Though hospitals have relatively limited time with each patient, their evidence-based practices and policies can support families to start and sustain breastfeeding during their hospital stay and build a foundation for good infant nutrition thereafter.

## Figures and Tables

**Table 1:** Measures of Maternity Care Practices and Policies Supportive of Breastfeeding—Maternity Practices in Infant Nutrition and Care (mPINC) Survey, United States, 2018–2022 ^a^.

Measure	Explanation	Survey Item(s)	Scoring
Domain: Immediate Postpartum Care			Mean of 4 measures ^b^
Immediate skin-to-skin contact	After vaginal delivery, percent of newborns who remain in uninterrupted skin-to-skin contact with their mothers immediately after birth if breastfeeding, until the first breastfeeding is completed, or if not breastfeeding, for at least 1 h.	C1_a1 C1_a2	100 = Most 70 = Many 30 = Some 0 = Few Items scored then averaged.
	After Cesarean delivery, percent of newborns who remain in uninterrupted skin-to-skin contact with their mothers as soon as the mother is responsive and alert if breastfeeding, until the first breastfeeding is completed, or if not breastfeeding, for at least 1 h.	C2_a1 C2_a2	100 = Most 70 = Many 30 = Some 0 = Few Items scored then averaged.
Transition to rooming-in	Percent of vaginally delivered newborns separated from their mothers before starting rooming-in.	C3	100 = Few 70 = Some 30 = Many 0 = Most OR Not an Option
Monitoring following birth	Percent of newborns who receive continuous observed monitoring throughout the first 2 h immediately following birth.	C5	100 = Most 70 = Many 30 = Some 0 = Few
Domain: Rooming-In			Mean of 3 measures
Rooming-in	Percent of newborns who stay in the room with their mothers for 24 h/day (not including separation for medical reasons).	C4_a1	100 = 80%+ 70 = 50–79% 30 = 20–49% 0 = <20%
Mother-infant separation	Indicates usual location of newborns during pediatric exams/rounds, hearing screening, pulse oximetry screening, routine labs/blood, draws/injections, and newborn bath.	C6_a1 C6_a2 C6_a4 C6_a5 C6_a6	100 = in mother’s room for all 5 situations 70 = removed from mother’s room for 1–2 situations 30 = removed from mother’s room for 3–4 situations 0 = removed from mother’s room for all 5 situations
Rooming-in safety	Indicates whether the hospital has a protocol requiring frequent observations, by nurses, of high-risk mother-infant dyads to ensure safety of the infant while they are together.	C7	100 = Yes 0 = No
Domain: Feeding Practices, Education, and Support ^c^			Mean of 4 measures
Glucose monitoring	Indicates whether the hospital performs routine blood glucose monitoring of full-term healthy newborns NOT at risk for hypoglycemia.	D5	100 = No 0 = Yes
Formula counseling for breastfeeding mothers	Frequency with which staff counsel breastfeeding mothers who request infant formula—about possible health consequences for their infant and the success of breastfeeding.	E3	100 = Almost always 70 = Often 30 = Sometimes 0 = Rarely
Feeding cues & pacifiers	Percent of breastfeeding mothers who are taught or shown how to recognize and respond to their newborn’s feeding cues, breastfeed as often and as long as their newborn wants, and understand the use and risks of artificial nipples and pacifiers.	E2_a1 E2_a5 E2_a7	100 = Most 70 = Many 30 = Some 0 = Few Items scored then averaged.
Identify/solve breastfeeding problems	Percent of breastfeeding mothers who are taught or shown how to position and latch their newborn for breastfeeding, assess effective breastfeeding by observing their newborn’s latch and the presence of audible swallowing, assess effective breastfeeding by observing their newborn’s elimination patterns, and hand express breast milk.	E2_a2 E2_a3 E2_a4 E2_a6	100 = Most 70 = Many 30 = Some 0 = Few Items scored then averaged.
Domain: Institutional Management			Mean of 5 measures
Nurse skill competency	Indicates which competency skills are required of nurses: - Placing and monitoring of the newborn skin-to-skin with the mother immediately following birth. - Assisting with effective newborn positioning and latch for breastfeeding. - Assessment of milk transfer during breastfeeding. - Assessment of maternal pain related to breastfeeding. - Teaching hand expression of breast milk. - Teaching safe formula preparation and feeding.	F4_a1 F4_a2 F4_a3 F4_a4 F4_a5 F4_a6	100 = 6 skills 80 = 5 skills 65 = 4 skills 50 = 3 skills 35 = 2 skills 20 = 1 skill 0 = 0 skills
Nurse competency assessment	Assesses whether formal assessment of clinical competency in breastfeeding support and lactation management is required of nurses.	F3	100 = Required at least every 2 years OR Less than every 2 years 0 = Not required
Documentation of exclusive breastfeeding	Indicated whether the hospital records/tracks exclusive breastfeeding throughout the entire hospitalization.	G1_a1	100 = Yes 0 = No
Acquisition of infant formula	Indicates how the hospital acquires infant formula.	G4_a1	100 = Pays fair market price 0 = Receives free OR Unknown/Unsure
Written policies	Indicates whether the hospital has a policy requiring the following: - Documentation of medical justification or informed consent for giving non-breast milk feedings to breastfed newborns. - Formal assessment of staff’s clinical competency in breastfeeding support. - Documentation of prenatal breastfeeding education. - Staff to teach mothers breastfeeding techniques AND staff to show mothers how to express milk. - Purchase of infant formula and related breast milk substitutes by the hospital at fair market value AND a policy prohibiting distribution of free infant formula, infant feeding products, and infant formula coupons. - Staff to provide mothers with resources for support after discharge. - Placement of all newborns skin-to-skin with their mother at birth or soon thereafter. - The option for mothers to room-in with their newborns.	G2_a1 G2_a2 G2_a4 G2_a5/G2_a6 G2_a8/G2_a12 G2_a9 G2_a7 G2_a11	100 = Yes 0 = No Final score is a mean of the 8 scores ^d^
Modified mPINC Score			Mean of 4 domains

^a^ Adapted from Scoring: Maternity Care Practices (https://www.cdc.gov/breastfeeding-data/mpinc/scoring.html (accessed on 25 November 2024)) and includes only the measures in the modified mPINC score for this analysis. Excludes the “Formula- feeding of breastfed infants” and “Formula preparation and feeding techniques” measures, and measures of discharge support. Domain scores were not calculated if half or more of the items in the section do not have a score. Total score was not calculated if any domain score is missing. ^b^ The domain score for hospitals with a valid skip for immediate skin-to-skin after Cesarean delivery was the mean of 3 items scored. ^c^ The Feeding Practices and Feeding Education domains are combined and rescored after removing the excluded measures to ensure that each domain has at least 3 component measures. ^d^ G2_a5 and G2_a6 as well as G2_a8 and G2_a12 are combined. Responses of Yes/Yes received a score of 100, other responses received a score of 0.

**Table 2:** Baseline Characteristics of Hospitals—Maternity Practices in Infant Nutrition and Care (mPINC) Survey, United States, 2018–2022 ^a^.

Characteristics at First Survey	n	%
Total	2109	100
Hospital Ownership Type		
Government/Military	119	5.6
Non-profit, private	1626	77.1
For profit, private	364	17.3
Hospital Size (annual births)		
<250	347	16.5
250–499	382	18.1
500–999	466	22.1
1000–1999	454	21.5
2000–4999	407	19.3
≥5000	53	2.5
Highest Level of Neonatal Care		
Level I: Well newborn nursery	842	40.0
Level II: Special care nursery	645	30.7
Level III: Neonatal Intensive Care Unit	525	25.0
Level IV: Regional Neonatal Intensive Care Unit	91	4.3
Region		
Northeast	195	9.3
Western	289	13.7
Mid-Atlantic	216	10.2
Midwest	492	23.3
Southwest	337	16.0
Mountain Plains	241	11.4
Southeast	339	16.1

^a^ Nested cohort of hospitals surveyed at least twice during the 2018, 2020, and 2022 cycles of the Maternity Practices in Infant Nutrition and Care (mPINC) survey.

**Table 3:** Exclusive Breastfeeding During the Delivery Hospitalization by Maternity Care Practices—Maternity Practices in Infant Nutrition and Care (mPINC) Survey, United States, 2018–2022 ^a^.

		In-Hospital Exclusive Breastfeeding ^b^					
		2018 (n = 1811)		2020 (n = 1949)		2022 (n = 1822)	
Practices	Score	n	% (SD)	n	% (SD)	n	% (SD)
Overall (0–100) ^c^	100	129	62.0 (18.2)	132	62.2 (20.0)	138	61.7 (19.2)
	80–99	839	59.8 (18.8)	1046	57.1 (19.1)	1044	54.7 (19.8)
	60–79	596	54.6 (20.6)	598	52.5 (21.8)	513	50.9 (21.3)
	<60	247	40.6 (20.5)	173	41.9 (22.4)	127	37.8 (22.3)
Immediate Postpartum Care (0–100) ^d^	100	612	60.9 (18.7)	719	60.2 (19.8)	703	58.3 (19.9)
	80–99	432	59.8 (19.4)	506	55.6 (19.5)	499	52.9 (20.2)
	60–79	490	53.2 (20.3)	495	52.4 (20.0)	409	50.5 (20.8)
	<60	276	41.8 (20.1)	228	40.6 (21.7)	211	40.2 (20.0)
Rooming-In (0–100) ^e^	100	352	61.5 (17.8)	494	60.0 (18.9)	465	56.1 (19.4)
	80–99	377	58.3 (19.7)	425	54.6 (19.5)	371	53.6 (21.1)
	60–79	495	56.4 (20.5)	574	54.2 (21.6)	558	52.9 (20.5)
	<60	584	49.8 (21.4)	455	49.6 (21.8)	427	49.1 (22.4)
Feeding Practices, Education, and Support (0–100) ^f^	100	807	60.3 (19.4)	900	59.8 (19.2)	859	56.7 (20.3)
	80–99	640	55.2 (19.8)	741	52.4 (20.3)	668	51.4 (20.4)
	60–79	285	47.8 (20.4)	253	46.5 (22.0)	255	47.0 (21.3)
	<60	79	39.5 (22.4)	55	40.0 (24.7)	40	37.5 (21.7)
Institutional Management (0–100) ^g^	100	382	60.9 (17.9)	426	58.1 (19.1)	467	55.6 (19.3)
	80–99	293	57.5 (20.4)	362	55.7 (20.1)	378	55.2 (20.0)
	60–79	550	53.8 (20.5)	558	53.0 (20.2)	593	51.1 (20.4)
	<60	586	52.9 (21.8)	603	53.3 (22.7)	384	50.5 (23.8)

^a^ Nested cohort of hospitals surveyed at least twice during the 2018, 2020, and 2022 cycles of the Maternity Practices in Infant Nutrition and Care (mPINC) survey. Scores and corresponding in-hospital exclusive breastfeeding rates are measured within the same year and do not represent within-hospital changes over time. A score of 100 indicates optimal adherence to best practices. ^b^ In-hospital exclusive breastfeeding is the percent of newborns who received only breast milk and no water or formula at any time during hospitalization as well as no glucose water or sucrose solution except during painful procedures. SD = standard deviation of mean percentage. ^c^ Overall score calculated as the average of each of the domain scores. Not directly aligned with published mPINC scores. ^d^ Score based on the frequency of immediate skin-to-skin contact, transition from delivery to rooming-in, and monitoring following birth. ^e^ Score based on the frequency of rooming-in, mother-infant separation, and rooming-in safety. ^f^ Score based on the frequency of glucose monitoring of healthy newborns not at risk of hypoglycemia, formula counseling for breastfeeding mothers, education on feeding cues and pacifiers, and education on how to identify and solve breastfeeding problems. ^g^ Score based on the frequency of requiring nurse skill competencies, nurse competency assessment, documentation of exclusive breastfeeding, acquisition of infant formula, and written policies supportive of breastfeeding.

## Data Availability

Data are available upon request by contacting mpinc@cdc.gov.

## Abbreviations

The following abbreviations are used in this manuscript:

CDC	U.S. Centers for Disease Control and Prevention
EBF	Exclusive breastfeeding
mPINC	Maternity Practices in Infant Nutrition and Care

## Appendix A

**Table A1:** Multi-Mediation Model (Path Analysis) of the Longitudinal Association of Maternity Care Practices and Policies With In-Hospital Exclusive Breastfeeding (EBF) Rates—Maternity Practices in Infant Nutrition and Care (mPINC) Survey, United States, 2018–2022 (n = 2109) ^a^.

Outcome	Mediator	Exposure	Unstandardized Estimate (95% CI) ^b^	Standardized Estimate (95% CI)
Direct Effects				
2022 EBF rate (p.p.) ^c^	-	2022 mPINC score (10 pts) ^d^	0.85 (0.40–1.30)	0.06 (0.03–0.08)
	-	Hospital size (100 births)	−0.03 (−0.07–0.01)	−0.02 (−0.05–0.01)
	-	2020 EBF rate (p.p.)	0.92 (0.88–0.96)	0.91 (0.88–0.93)
2020 EBF rate (p.p.)	-	2020 mPINC score (10 pts)	0.79 (0.39–1.19)	0.06 (0.03–0.08)
	-	Hospital size (100 births)	−0.07 (−0.11–−0.03)	−0.05 (−0.08–−0.02)
	-	2018 EBF rate (p.p.)	0.80 (0.77–0.83)	0.80 (0.78–0.82)
2018 EBF rate (p.p.)	-	2018 mPINC score (10 pts)	4.86 (4.30–5.41)	0.38 (0.34–0.42)
	-	Hospital size (100 births)	−0.24 (−0.30–−0.18)	−0.17 (−0.21–−0.13)
2022 mPINC score (pts)	-	2020 mPINC score (10 pts)	0.83 (0.77–0.88)	0.89 (0.84–0.93)
	-	Hospital size (100 births)	0.00 (0.00–0.01)	0.02 (−0.02–0.06)
2020 mPINC score (pts)	-	2018 mPINC score (10 pts)	0.60 (0.57–0.63)	0.66 (0.64–0.69)
	-	Hospital size (100 births)	0.01 (0.00–0.01)	0.08 (0.04–0.11)
Indirect Effects				
2022 EBF rate (p.p.)	2020 and 2022 mPINC score	2018 mPINC score (10 pts)	0.42 (0.20–0.65)	0.03 (0.01–0.05)
2022 EBF rate (p.p.)	2018 and 2020 EBF rate	2018 mPINC score (10 pts)	3.58 (3.14–4.01)	0.27 (0.24–0.30)
2022 EBF rate (p.p.)	2022 mPINC score and 2020 EBF rate	2018 mPINC score (10 pts)	0.44 (0.21–0.66)	0.03 (0.02–0.05)
Total Effects				
2022 EBF rate (p.p.)	-	2018 mPINC score (10 pts)	4.44 (3.96–4.92)	0.34 (0.30–0.37)

Note: CI, confidence interval; p.p., percentage points; pts, points. Model Fit Statistics: Chi-square(6 df) = 57.3 (*p* < 0.05); root mean square error of approximation = 0.064; standardized root mean square residual = 0.021; comparative fit index = 0.992; Tucker-Lewis index = 0.973. ^a^ Nested cohort of hospitals surveyed at least twice during the 2018, 2020, and 2022 cycles of the Maternity Practices in Infant Nutrition and Care (mPINC) survey. ^b^ Unstandardized estimates are in units of the dependent variable. ^c^ In-hospital exclusive breastfeeding is the percent of newborns who received only breast milk and no water or formula at any time during hospitalization as well as no glucose water or sucrose solution except during painful procedures. ^d^ Modified mPINC score calculated as the average of 4 domain scores. Not directly aligned with published mPINC scores. Domains include (a) immediate skin-to-skin contact, transition from delivery to rooming-in, and monitoring following birth; (b) rooming-in, mother-infant separation, and rooming-in safety; (c) glucose monitoring of healthy newborns not at risk of hypoglycemia, formula counseling for breastfeeding mothers, education on feeding cues and pacifiers, and education on how to identify and solve breastfeeding problems; (d) requiring nurse skill competencies, nurse competency assessment, documentation of exclusive breastfeeding, acquisition of infant formula, and written policies supportive of breastfeeding.

**Table A2:** Structural Equation Model of Maternity Care Practices and Policies and In-Hospital Exclusive Breastfeeding (EBF) Rates—Maternity Practices in Infant Nutrition and Care (mPINC) Survey, United States, 2018–2022 (n = 2109) ^a^.

Dependent Variable	Mediator	Independent Variable	Standardized to Latent Variables Estimate (95% CI) ^b^	Standardized Estimate (95% CI)
Latent Factor Loadings ^c^				
Immediate skin-to-skin contact (≥80% vs. <80%)	-	Maternity care practices (SD)	0.24 (0.22–0.26)	0.48 (0.45–0.52)
Transition to rooming-in (≥80% vs. <80%)	-		0.09 (0.07–0.10)	0.25 (0.22–0.29)
Monitoring following birth (≥80% vs. <80%)	-		0.09 (0.08–0.11)	0.20 (0.17–0.24)
Rooming-in (≥80% vs. <80%)	-		0.14 (0.13–0.16)	0.33 (0.29–0.36)
Mother-infant separation (mother’s room for all vs. less)	-		0.23 (0.21–0.25)	0.53 (0.49–0.56)
Rooming-in safety (protocol vs. no protocol)	-		0.13 (0.11–0.14)	0.28 (0.24–0.31)
Glucose monitoring (no vs. yes)	-		0.03 (0.02–0.05)	0.12 (0.08–0.16)
Formula counseling for breastfeeding mothers (almost always vs. less often)	-		0.25 (0.23–0.27)	0.51 (0.48–0.55)
Feeding cues & pacifiers (≥80% vs. <80%)	-		0.20 (0.18–0.21)	0.45 (0.41–0.48)
Identify/solve breastfeeding problems (≥80% vs. <80%)	-		0.23 (0.21–0.24)	0.48 (0.45–0.52)
Nurse skill competency (6 skills vs. fewer)	-		0.28 (0.26–0.30)	0.58 (0.55–0.61)
Nurse competency assessment (every 2 years vs. less often)	-		0.21 (0.19–0.23)	0.43 (0.40–0.47)
Documentation of exclusive breastfeeding (yes vs. no)	-		0.07 (0.06–0.08)	0.28 (0.24–0.32)
Acquisition of infant formula (pays vs. free)	-		0.31 (0.29–0.33)	0.64 (0.61–0.67)
Written policies (yes vs. no)	-		0.31 (0.29–0.33)	0.67 (0.64–0.70)
Direct Effects				
2022 EBF rate (p.p.) ^d^	-	2022 maternity care practices (SD)	0.64 (−0.09–1.36)	0.03 (0.00–0.06)
	-	Hospital size (100 births)	−0.02 (−0.06–0.02)	−0.01 (−0.04–0.02)
	-	2020 EBF rate (p.p.)	0.93 (0.89–0.97)	0.91 (0.89–0.94)
2020 EBF rate (p.p.)	-	2020 maternity care practices (SD)	0.94 (0.28–1.60)	0.04 (0.01–0.08)
	-	Hospital size (100 births)	−0.06 (−0.10–−0.02)	−0.05 (−0.07–−0.02)
	-	2018 EBF rate (p.p.)	0.80 (0.78–0.83)	0.80 (0.78–0.82)
2018 EBF rate (p.p.)	-	2018 maternity care practices (SD)	6.86 (5.84–7.88)	0.33 (0.28–0.37)
	-	Hospital size (100 births)	−0.21 (−0.27–−0.15)	−0.15 (−0.19–−0.11)
2022 maternity care practices (pts)	-	2020 maternity care practices (SD)	0.91 (0.89–0.94)	0.91 (0.89–0.94)
	-	Hospital size (100 births)	0.00 (0.00–0.00)	0.02 (−0.02–0.06)
2020 maternity care practices (pts)	-	2018 maternity care practices (SD)	0.87 (0.84–0.89)	0.87 (0.84–0.89)
	-	Hospital size (100 births)	0.01 (0.00–0.01)	0.08 (0.04–0.11)
Indirect Effects				
2022 EBF rate (p.p.)	2020 and 2022 maternity care practices	2018 maternity care practices (SD)	0.50 (−0.07–1.08)	0.02 (0.00–0.05)
2022 EBF rate (p.p.)	2018 and 2020 EBF rate	2018 maternity care practices (SD)	5.13 (4.33–5.92)	0.24 (0.21–0.27)
2022 EBF rate (p.p.)	2020 maternity care practices and 2020 EBF rate	2018 maternity care practices (SD)	0.76 (0.22–1.29)	0.04 (0.01–0.06)
Total Effects				
2022 EBF rate (p.p.)	-	2018 maternity care practices (SD)	6.39 (5.43–7.35)	0.30 (0.26–0.34)

CI, confidence interval; SD, standard deviation; p.p., percentage points; pts, points. Only latent variables are standardized. Estimates are in units of the dependent variable. Model Fit Statistics: Chi-square(1011 df) = 3215.7 (*p* < 0.001); root mean square error of approximation = 0.032; standardized root mean square residual = 0.050; comparative fit index = 0.923; Tucker-Lewis index = 0.911. ^a^ Nested cohort of hospitals surveyed at least twice during the 2018, 2020, and 2022 cycles of the Maternity Practices in Infant Nutrition and Care (mPINC) survey. ^b^ Estimates standardized to latent variables are in units of the dependent variable. ^c^ Factor loadings were constrained to be equivalent across the survey cycles. ^d^ In-hospital exclusive breastfeeding is the percent of newborns who received only breast milk and no water or formula at any time during hospitalization as well as no glucose water or sucrose solution except during painful procedures.
